# Supplementary material for: Collaborative intelligence in AI: Evaluating the performance of a council of AIs on the USMLE
Source: PLOS Digit Health. 2025 Oct 9;4(10):e0000787. doi: 10.1371/journal.pdig.0000787 (PMC12510544; doi:10.1371/journal.pdig.0000787)
Supplement: S2 File — (ZIP) [file pdig.0000787.s002.zip › S2 Data Raw data files containing unprocessed question inputs and LLM outputs including transcripts of deliberations/STEP3_Question-78-1_Type-MC-NJ.docx]

**Facilitator** **[Round 1]:** A 54-year-old woman comes to the office because she has had intermittent shooting pain over her right cheek and jaw during the past 3 weeks. Each episode of pain lasts for 1 second or less. The pain is often triggered by cold air, chewing, tactile stimulation, and brushing her teeth. She has had no trauma to the face or head. Medical history is remarkable for tension headaches, obesity, and gastric bypass surgery. She is 165 cm (5 ft 5 in) tall and weighs 62 kg (137 lb); BMI is 23 kg/m². Vital signs are normal. The patient cannot tolerate touch over the right side of the face. There is no facial weakness or loss of sensation. The remainder of the physical examination shows no abnormalities. CT scan of the head with and without contrast shows no abnormalities. Which of the following is the most appropriate pharmacotherapy at this time? (A) Carbamazepine (B) Lamotrigine (C) Levetiracetam (D) Topiramate (E) Zonisamide

**AI_E:** 1) RESTATING THE QUESTION: This inquiry is concerning a 54-year-old woman who is experiencing sporadic, sharp pains in her right cheek and jaw, which are triggered by factors such as cold air, chewing, brushing her teeth, and tactile stimulation. The episodes are noted to last less than one second. A physical examination reveals intolerance to touch on the right side of her face, however, the patient has no loss of sensation and shows no facial weakness. Her CT scan was uneventful. She has a history of tension headaches, obesity, and gastric bypass surgery. The patient's BMI is 23 kg/m² and her vital signs are all normal. The question is asking which pharmacotherapy would be most appropriate for her condition at the current time. 2) PLANNING: We are assuming that the woman's symptoms: the sharp, intermittent pain in her face and jaw, the intolerance to touch, and the triggers are related to a specific neurological condition. We can question whether these signs point towards Trigeminal Neuralgia, a chronic pain disorder affecting the trigeminal nerve, which carries sensation from your face to your brain. In this condition, even mild stimulation of the face might trigger a jolt of excruciating pain. It's critical to establish and prioritize this assumption for clinical decision-making. 3) CLINICAL REASONING: The given clinical picture of sudden, sharp, severe facial pain triggered by normal activities like chewing or brushing teeth is very indicative of Trigeminal Neuralgia. The absence of trauma, normal neurologic examination, and a normal CT head scan further supports this assumption, as Trigeminal Neuralgia typically does not cause any objective signs and imaging is usually normal. Hence, we are dealing with a case of classical Trigeminal Neuralgia. 4) CONCLUDING: Trigeminal Neuralgia is typically managed with pharmacotherapy in the form of class of medications called anticonvulsant drugs. Among the options provided Carbamazepine is considered as first-line therapy as it has been extensively studied for this indication and has a long track record of effectiveness in managing Trigeminal Neuralgia. 5) RESPONSE: Therefore, the most appropriate pharmacotherapy for this patient at this time, based on her clinical presentation, is likely Carbamazepine.

**AI_B:** 1) RESTATING THE QUESTION: A middle-aged woman with a past medical history of tension headaches, obesity, and gastric bypass surgery is experiencing brief, intermittent, sharp pains in the right side of her face, triggered by activities such as exposure to cold air, chewing, tactile stimulation, and brushing her teeth. She is averse to touch over the right side of her face. However, there is no loss of sensation or facial weakness. Examination, vital signs, and head CT scans are otherwise normal. We are tasked with choosing the most suitable pharmacotherapy for this condition. 2) PLANNING: The primary assumption here is that the woman's symptoms are suggestive of trigeminal neuralgia (TN), a condition caused by irritation of the trigeminal nerve, which results in sharp, brief, episodic facial pain that can be triggered by trivial stimuli. We could also potentially consider other causes of facial pain like dental pain, migraine, or post-herpetic neuralgia. However, given the abrupt onset of pain, localisation to the distribution of the trigeminal nerve, absence of trauma or infection, and triggering by trivial stimuli, TN appears to be the most likely diagnosis. 3) CLINICAL REASONING: In trigeminal neuralgia, neural hyperactivity in the trigeminal nerve is caused by its compression, inflammation, or demyelination, leading to paroxysmal facial pain triggered by small stimuli. Pharmacotherapy usually targets decreasing neural hyperactivity. This reasoning aligns with our primary assumption. Among drugs that can reduce neural hyperactivity, carbamazepine and oxcarbazepine are first-line choices. 4) CONCLUDING: Given the clinical scenario, this woman's facial pain probably arises from trigeminal neuralgia. The first line of treatment is carbamazepine or oxcarbazepine. These agents normalize the discharge of sodium-dependent action potentials in neurons, which helps in controlling acute symptoms of pain and preventing further neuralgic episodes. 5) RESPONSE: The most appropriate pharmacotherapy at this time for this woman is Carbamazepine (option A).

**AI_A:** RESTATING THE QUESTION: This 54-year-old woman presents with intermittent, intense, shooting pain in her right cheek and jaw over the past three weeks, triggered by various stimulations like cold air, chewing, and brushing her teeth. The pain episodes are brief, lasting 1 second or less. Despite no trauma or observable physical or neural abnormalities, she cannot tolerate touch on the affected area. She has a medical history of tension headaches, obesity, and gastric bypass surgery. We're being asked to identify the suitable pharmacological treatment from a given list. PLANNING: A key assumption here could be that her symptoms suggest a neurological disorder, not caused by any apparent trauma or physical abnormalities as ruled out by the CT scan. Another assumption would be that these symptoms could be indicative of a condition like trigeminal neuralgia (TN), where patients experience intense, episodic facial pain. An alternative assumption to debate is whether this could be linked to other conditions mentioned in her medical history like tension headaches or any secondary consequences of obesity and gastric bypass surgery. CLINICAL REASONING: Considering the intense, brief and sporadic nature of the pain in the facial region, which is characteristic of TN, and that her pain is associated with normal activities like chewing and brushing, which can generally trigger the symptoms of TN, these points towards this assumption. TN is a kind of neuropathic pain disorder involving the trigeminal nerve and generally affects adults older than 50. Obesity and gastric bypass surgery do not typically present with these symptoms. Tension headaches could have been a plausible assumption, but the classic features of TN distinguish it. CONCLUDING: TN is a clinical diagnosis made on the basis of typical symptoms (as in this patient), and additional testing (eg, neuroimaging) is not routinely required unless there are atypical features such as sensory loss, which were not present in the patient. The first-line treatment for TN is an antiseizure medication, carbamazepine, which stabilizes voltage-gated sodium channels in their inactive configuration, thus diminishing the frequency of neuronal firing. RESPONSE: In the given scenario, given the symptoms and their alignment with the characteristics of trigeminal neuralgia, the most appropriate medication would be (A) Carbamazepine, serving as the first-line treatment effectively suppressing TN attacks.

**AI_D:** RESTATING THE QUESTION: The scenario describes a 54-year-old woman who has been consistently experiencing sharp, fleeting pain on the right side of her face, particularly in the areas of her cheek and jaw, over the past few weeks. The pain appears to be triggered by certain stimuli, including cold air, chewing, touch, and teeth brushing. The patient has a medical history of tension headaches, obesity, and gastric bypass surgery. She is of normal weight and height, and her vital signs are reported to be normal. A physical examination reveals a hypersensitivity to touch on the right side of her face, though there is no apparent weakness or loss of sensation. A CT scan has not shown any abnormalities. The task at hand is to identify the most appropriate pharmacological treatment for her condition from the options given: Carbamazepine, Lamotrigine, Levetiracetam, Topiramate, or Zonisamide. PLANNING: The central assumption here is that the woman's symptoms are indicative of a specific neurological condition. An alternate assumption could be that her symptoms are related to her past medical history. However, given her symptom pattern, especially the sharp, fleeting pain triggered by stimuli such as chewing or touch, the most plausible clinical assumption is trigeminal neuralgia, a condition often characterized by such acute facial pain. Thus, pharmacotherapy should be aligned in that context. An evaluation of the proposed pharmacotherapies demonstrates that all are antiepileptic drugs (AEDs), although their mechanisms and indication vary. CLINICAL REASONING: When considering the nature of the patient's symptoms and the potential diagnosis of trigeminal neuralgia, the identification of the most appropriate pharmacotherapy lies in understanding the efficacy and indication of each drug listed. Carbamazepine is a sodium channel blocker that is commonly indicated for the treatment of trigeminal neuralgia by reducing hyperactive firing of the trigeminal nerve. Lamotrigine, Levetiracetam, Topiramate, and Zonisamide, whilst being AEDs, are not considered first-line treatments for trigeminal neuralgia. CONCLUDING: Given the suggestive features of trigeminal neuralgia in the patient's presentation and the drug efficacy profile, the most appropriate pharmacotherapy to recommend would be the one recognized as the first-line treatment for trigeminal neuralgia. RESPONSE: The most appropriate pharmacotherapy for this patient at this time is (A) Carbamazepine.

**AI_C:** (1) RESTATING THE QUESTION: The 54-year-old woman, who has a medical history of tension headaches, obesity and gastric bypass surgery, is presenting symptoms of a sharp and rapid onset pain in her right cheek and jaw that comes and goes. Triggers include cold air, activities like chewing, touch and brushing her teeth. She has not experienced any trauma to her face or head. Physical examination reveals hypersensitivity on the right side of her face, however no abnormalities, including facial weakness or loss of sensation, are discovered. A head CT scan with contrast does not show any abnormalities. The question before us now is: what medication would be the most suitable for her condition among carbamazepine, lamotrigine, levetiracetam, topiramate, and zonisamide? (2) PLANNING: Considering the symptoms and signs, this patient seems to be suffering from trigeminal neuralgia. It is caused by irritation or damage to the trigeminal nerve, or cranial nerve V, which sends impulses from the brain to the face. The pain is characteristic: severe, sharp, sudden, and brief. Common triggers are cold air, chewing, and brush of touch across the face. The first line of treatment for this condition is usually anticonvulsant medication since it helps reduce the nerve's irritability. Of the options provided, carbamazepine is the one typically used for initial therapy in classic trigeminal neuralgia. (3) CLINICAL REASONING: Trigeminal neuralgia (TN) is characterized by intermittent intense pain in the areas supplied by the trigeminal nerve often provoked by ordinary stimuli such as touching, speaking, or moving the face. This patient’s symptoms of severe, transient facial pain triggered by nonpainful stimuli such as brushing teeth are characteristic of TN. While the CT scan appears normal, trigeminal neuralgia is often idiopathic, with imaging studies being negative in a majority of cases. Carbamazepine is the drug of choice for the treatment of trigeminal neuralgia. It stabilizes the sodium channels of hyperexcited neurons, making them less excitable, thus reducing symptoms. (4) CONCLUDING: In connection with the symptoms described by the patient, the chronic course of the disease, episodic facial pain triggered by various stimuli and normal CT scan findings, we conclude that the patient most likely has trigeminal neuralgia. In light of the drugs provided, the standard treatment for this condition is the anticonvulsant, carbamazepine. (5) RESPONSE: The most appropriate pharmacotherapy for this patient at this time is (A) Carbamazepine.

**Facilitator** The consensus statement synthesizes the responses of AI_A, AI_B, AI_C, AI_D, and AI_E as follows: The 54-year-old woman's symptoms of intermittent, intense, shooting pain in her right cheek and jaw, triggered by various stimulations like cold air, chewing, and brushing her teeth, suggest a neurological disorder. The pain episodes are brief, lasting 1 second or less, and she cannot tolerate touch on the affected area. Despite no trauma or observable physical or neural abnormalities, her symptoms could be indicative of a condition like trigeminal neuralgia (TN), where patients experience intense, episodic facial pain. The intense, brief, and sporadic nature of the pain in the facial region, which is characteristic of TN, and the fact that her pain is associated with normal activities like chewing and brushing, which can generally trigger the symptoms of TN, point towards this assumption. TN is a kind of neuropathic pain disorder involving the trigeminal nerve and generally affects adults older than 50. TN is a clinical diagnosis made on the basis of typical symptoms, and additional testing is not routinely required unless there are atypical features such as sensory loss, which were not present in the patient. The first-line treatment for TN is an antiseizure medication, carbamazepine, which stabilizes voltage-gated sodium channels in their inactive configuration, thus diminishing the frequency of neuronal firing. Given the symptoms and their alignment with the characteristics of trigeminal neuralgia, the most appropriate medication would be Carbamazepine, serving as the first-line treatment effectively suppressing TN attacks.
